# Supplementary material for: Anomeric memory of the glycosidic bond upon fragmentation and its consequences for carbohydrate sequencing
Source: Nat Commun. 2017 Oct 17;8:973. doi: 10.1038/s41467-017-01179-y (PMC5645458; doi:10.1038/s41467-017-01179-y)
Supplement: Supplementary file 1 — Supplementary Information [file 41467_2017_1179_MOESM1_ESM.pdf]

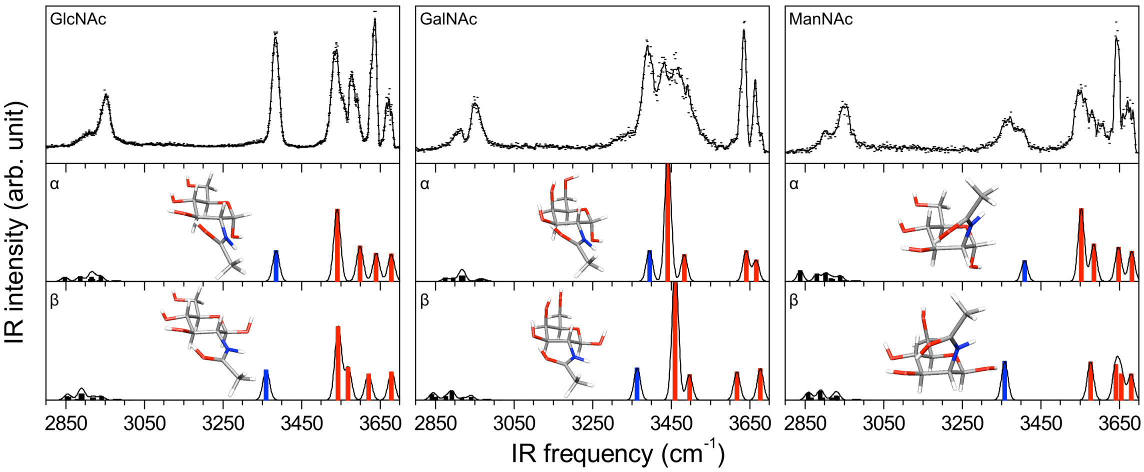

Supplementary Fig. 1. Conformational analysis of N-acetylhexosamines*. The vibrational spectra of the lowest energy structures of both anomers of protonated GlcNAc, GalNAc and ManNAc are obtained by Density Functional Theory, as described above, and match the experimental spectra. A colour code is used to represent the OH (red), NH (blue) and CH (black) elongation modes. In all cases, the lowest energy structures obtained by quantum chemistry calculations are $^4C^1$ chairs. OH6 and OH1 modes have a relatively fixed frequency (around 3680 cm^{-1} and 3640 cm^{-1}, respectively) regardless of the isomer. In contrast, the frequencies of OH3 and OH4 range from 3400 cm^{-1} to 3650 cm^{-1} are strongly isomer-dependent, which explains the sensitivity of the IR fingerprint to the nature of the monosaccharide. Interestingly, theory predicts that pairs of anomers have the same stable conformation. Yet their OH3 and OH4 modes as well as the NH mode of the N-acetyl group are anomer-dependent, providing distinctive theoretical signatures for individual anomers. Here, the N-acetylhexosamine standards consist of natural anomeric mixtures. Therefore, the theoretical signatures of both anomers must be taken into account to reproduce the experimental signature.*

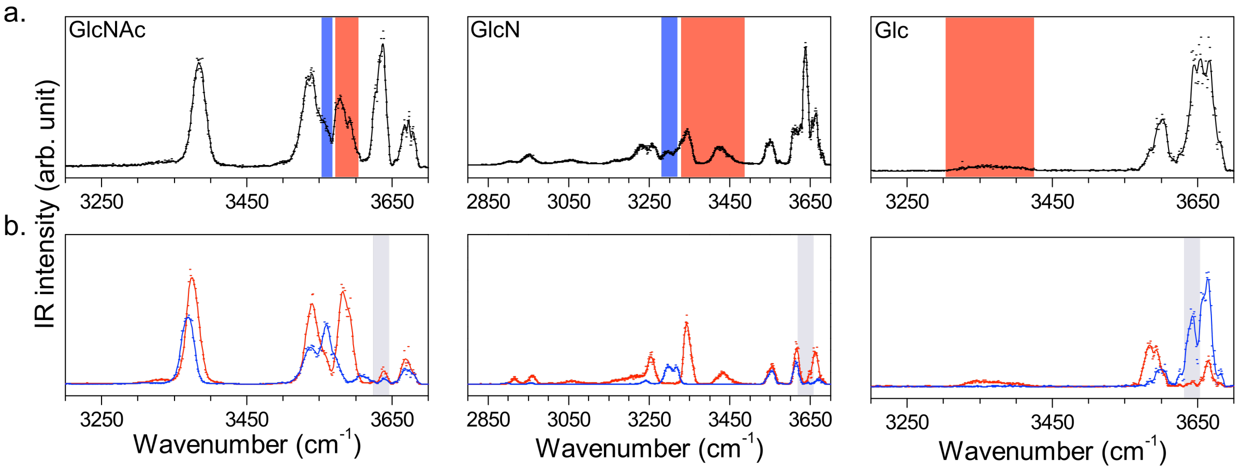

Supplementary Fig. 2. Identification of the diagnostic features of alpha and beta anomers. *(a) As predicted by theory (Supplementary Fig. 1) individual anomers of monosaccharide standards have distinctive signatures, both being present in relative amounts in the spectrum of natural anomeric mixtures. This property is exploited to identify the contributions of both anomers present in the spectrum of natural samples. The diagnostic features of alpha and beta anomers thus identified are shaded in red and blue, respectively. (b) Additionally, the reference signatures of individual anomers chemically blocked by methylation at OH1 (alpha anomer in red, beta anomer in blue) are superimposed and compared with the spectrum of the natural mixture. Due to the methylation of the reducing hydroxyl, the intense OH1 modes normally observed around 3640 cm*$^{-1}$ *are absent (shaded in grey). Apart from this exception, all features of the methyl-blocked species (b) are found in the spectrum of the natural anomeric mixture (a), which validates that they have the same conformation. Hence, after discarding the OH1 spectral range, the spectra of the methylated standards can be used as references to identify diagnostic features of the anomers in the spectrum of the natural sample.*

Supplementary methods

Sample preparation.

Monosaccharide standards: monosaccharides standards were purchased from Sigma Aldrich or carbosynth Ltd (UK) and used without further purification.
Disaccharide standards: GlcNAc β(1→6) GlcNAc and GlcN β(1→4) GlcNAc were prepared as reported earlier by Defaye et al. and Drouillard et al. respectively[1,2]
Glc α(1→4) GlcNAc, Glc β(1→4) GlcNAc, Man α(1→4) GlcNAc and Gal α(1→4) GlcNAc, were prepared according to the protocols of Both et al.[3] and Gray et al.[4]
Other disaccharides were purchased from Sigma Aldrich, carbosynth Ltd (UK) or Dextra Laboratories Ltd. (UK).
Chito-oligosaccharide sample: It was prepared as reported by Chambon et al.[5] Note that no experimental evidence of the position of the enzymatic deacetylation was available.

Computational chemistry.

To obtain the conformers of tmonosaccharides, we used the following procedure: First of all, we employed the molecular dynamic to produce geometries. This step consisted in 3 parts, first the initial geometry was heated at a temperature of 3000 K for 1 ps, allowing exhaustive exploration of the potential energy surface; then the system was equilibrated for an other ps; finally 1000 geometries were captured with 10 fs intervals for 10 ps. During this step, the forces were computed by the semi-empirical potential PM7[6] carried out using MOPAC2012.[7] We chose PM7 because it was developed to correct the hydrogen bonding treatment. The velocity-Verlet algorithm[8] was used to calculate the positions and the velocities.
The initial geometries were then optimised with PM7 and duplicates were discarded based on geometric and energetic parameters (two structures are considered identical if $\Delta E < 0.5$ kcal/mol and Δd for bonded and non-bonded atoms is inferior to 0.2 Å).
In the second step, we reoptimised the candidate geometries with Density functional Theory (DFT) using the functional B3LYP[9-11] and the basis set 6-31G* [12-13]. Once again we eliminated identical geometries.
For the final step, the geometries were further optimised and the harmonic vibrational frequencies were calculated using DFT with CAM-B3LYP[14] and a higher basis set 6-311+ +G(2df,2pd). The mode analysis was carried out with Gabedit.[15]
All the DFT calculations were done by using the Gaussian 09 suite.[16] The geometry optimisation was done using tight convergence criteria.
We compared the theoretical spectra with experimental ones after scaling the harmonic frequencies. The scaling factor employed to best match the experiment was 0,947.
The total energies of the final geometries were computed at a MP2 level using the Pople basis set 6-311++G(3df,3pd). All these single-point calculations were run in the 3.0.2 version of ORCA.[17]
Important note: at this level of theory, the intensity of H-bonded modes is overestimated and their experimental broadening is not reproduced. Yet, they can be assessed with confidence based on their frequency and the correlation between the experimental broadening and the H-bonded character observed in the mode analysis.
A example of simulation of the vibrational frequencies is shown in Supplementary Fig. 1 for lowest energy conformers of three N-acetylhexosamines.MS/IR analysis

Supplementary References

1. Defaye, J., Gadelle, A & Pedersen, C. Hydrogen fluoride-catalyzed formation of glycosides. Preparation of methyl 2-acetamido-2-deoxy-β-d-gluco- and -β-d-galacto-pyranosides, and of β-(1→6)-linked 2-acetamido-2-deoxy-d-gluco- and -d-galacto-pyranosyl oligosaccharides. *Carbohyd. Res*. **186**, 177-188 (1989).
2. Drouillard, S., Armand, S., Davies, G.J., Vorgias, C. & Henrissat, B. Serratia marcescens chitobiase is a retaining glycosidase utilizing substrate acetamido group participation. *Biochem. J*. **328**, 945-949 (1997).
3. Both, P. et al. Discrimination of epimeric glycans and glycopeptides using IM-MS and its potential for carbohydrate sequencing. *Nature Chem*. **6**, 65-74 (2014).
4. Gray, C. J. et al. Bottom-Up Elucidation of Glycosidic Bond Stereochemistry. *Anal. Chem*. **89**, 4540-4549 (2017).
5. Chambon, R. et al. Efficient chemoenzymatic synthesis of lipo-chitinoligosaccharides plant growth promotors. *Green Chem.* **17**, 3923-3930 (2015).
6. Stewart, J. J. *J.* Optimization of parameters for semiempirical methods VI: more modifications to the NDDO approximations and re-optimization of parameters. *Mol. Model.* **19**, 1-32 (2013).
7. MOPAC2012, J.J.P. Stewart, Stewart Computational Chemistry; Colorado Springs, CO, USA.
8. Swope, W. C., Andersen, H. C., Berens, P. H. & Wilson, K. R. A computer simulation method for the calculation of equilibrium constants for the formation of physical clusters of molecules: Application to small water clusters. *J. Chem. Phys.* **76,** 637–649 (1982).
9. Lee, C., Yang, W. & Parr, R. G. Development of the Colle-Salvetti correlation-energy formula into a functional of the electron density. *Phys. Rev. B* **37**, 785 (1988).
10. Vosko, S. H., Wilk, L. & Nusair, M. Accurate spin-dependent electron liquid correlation energies for local spin density calculations: a critical analysis. *Can. J. Phys.* **58**, 1200-1211 (1980).
11. Stephens, P. J., Devlin., F. J., Chabalowski, C. F. & Frisch, M. J. Ab Initio Calculation of Vibrational Absorption and Circular Dichroism Spectra Using Density Functional Force Fields. *J. Phys. Chem.* **98**, 11623–11627 (1994).
12. Hehre, W. J., Ditchfield, R. & Pople, J. A. Self—Consistent Molecular Orbital Methods. XII. Further Extensions of Gaussian—Type Basis Sets for Use in Molecular Orbital Studies of Organic Molecules. *J. Chem. Phys.* **56**, 2257-2261 (1972).
13. Krishnan, R., Binkley, J. S., Seeger, R. & Pople, J. A. Self-consistent molecular orbital methods. XX. A basis set for correlated wave functions *J. Chem. Phys.* **72**, 650–654 (1980).
14. Yanai, T., Tew, D. P. & Handy, N. C.A new hybrid exchange–correlation functional using the Coulomb-attenuating method (CAM-B3LYP). *Chem. Phys. Lett.* **393**, 51–57 (2004).
15. Allouche, A-R. Gabedit—A graphical user interface for computational chemistry softwares *J. Comput. Chem*. **32**, 174-182 (2011).
16. Frisch, M. J. *et al.* Gaussian 09, Gaussian, Inc., Wallingford, CT, USA, 2009.
17. Neese, F. The ORCA program system. *Wiley Interdiscip. Rev. Comput. Mol. Sci.* **2**, 73–78 (2012).
